# Evaluation of polymorphisms in microRNA‐binding sites and pancreatic cancer risk in Chinese population

**DOI:** 10.1111/jcmm.14906

**Published:** 2019-12-27

**Authors:** Juntao Ke, Xiating Peng, Shufang Mei, Jianbo Tian, Pingting Ying, Nan Yang, Xiaoyang Wang, Danyi Zou, Yang Yang, Ying Zhu, Yajie Gong, Jing Gong, Rong Zhong, Jiang Chang, Zemin Fang, Xiaoping Miao

**Affiliations:** ^1^ State Key Laboratory of Environment Health (Incubation) Key Laboratory of Environment & Health (Ministry of Education) Ministry of Environmental Protection Key Laboratory of Environment and Health (Wuhan) Department of Epidemiology and Biostatistics, School of Public Health Tongji Medical College Huazhong University of Science and Technology Wuhan China; ^2^ College of Informatics Huazhong Agricultural University Wuhan China; ^3^ Division of Cardiothoracic and Vascular Surgery Tongji Hospital Tongji Medical College Huazhong University of Science and Technology Wuhan China

**Keywords:** Chinese population, genome‐wide screening, microRNA‐binding sites, pancreatic cancer, polymorphisms

## Abstract

As promising biomarkers and therapy targets, microRNAs (miRNAs) are involved in various physiological and tumorigenic processes. Genetic variants in miRNA‐binding sites can lead to dysfunction of miRNAs and contribute to disease. However, systematic investigation of the miRNA‐related single nucleotide polymorphisms (SNPs) for pancreatic cancer (PC) risk remains elusive. We performed integrative bioinformatics analyses to select 31 SNPs located in miRNA‐target binding sites using the miRNASNP v2.0, a solid database providing miRNA‐related SNPs for genetic research, and investigated their associations with risk of PC in two large case‐control studies totally including 1847 cases and 5713 controls. We observed that the SNP rs3802266 is significantly associated with increased risk of PC (odds ratio (OR) = 1.21, 95% confidence intervals (CI) = 1.11‐1.31, *P* = 1.29E‐05). Following luciferase reporter gene assays show that rs3802266‐G creates a stronger binding site for miR‐181a‐2‐3p in 3′ untranslated region (3′UTR) of the gene *ZHX2*. Expression quantitative trait loci (eQTL) analysis suggests that *ZHX2* expression is lower in individuals carrying rs3802266‐G with increased PC risk. In conclusion, our findings highlight the involvement of miRNA‐binding SNPs in PC susceptibility and provide new clues for PC carcinogenesis.

## 1 INTRODUCTION

Pancreatic cancer (PC) is the twelfth most common cancer around the world^1^ and is one of the most lethal human cancers with a rather low 5‐year survival rate of 5%.^2^, ^3^ In China, there are estimated over 90 000 new cases and nearly 80 000 related deaths in 2015.^4^ Chronic pancreatitis, type 2 diabetes, obesity and cigarette smoking have been established as PC risk factors. Meanwhile, the genetic pathogenesis of PC is still unclear.^5^, ^6^

Over the last decade, genome‐wide association studies (GWASs) have identified multiple PC‐associated chromosome loci.^7^ For Chinese population, the first GWAS located 6 susceptibility loci (5p13.1, 10q26.11, 13q22.1, 21q21.3, 21q22.3 and 22q13.32)^8^ and a subsequent investigation further indicated another locus 17q24.3 containing *LINC00673* contributing to PC risk.^9^ Recently, our group performed a exome‐wide association study (EWAS) and discovered three new associated regions (19p13.12, 8p21.3 and 2p24.1).^10^ However, the single nucleotide polymorphisms (SNPs) identified by GWASs and EWASs could only explain a minor portion of heritability,^11^, ^12^ and the missing heritability of PC remains to be dissected.^13^

MicroRNAs (miRNAs) are endogenous small non‐coding RNAs constituting of about 22 nucleotides,^14^, ^15^ and they could inhibit mRNA translation and induce mRNA decay via base‐pairing binding to the 3′ UTR of target mRNAs.^16^, ^17^ As valuable biomarkers and promising therapeutic targets, miRNAs are found involved in multiple physiological and tumorigenic processes.^18^ Meanwhile, SNPs in miRNAs' target binding sites could impact the interactions between miRNAs and target genes, functioning as regulatory variants for various phenotypes and diseases.^19^ Previously, we upgraded a widely used online database called miRNASNP v2.0,^20^ and successfully applied it to locate a functional SNP rs1062044 affecting miR‐423‐5p binding to the gene *LAMC1* in colorectal cancer susceptibility locus 1q25.3.^21^ Still, systematic investigation on the miRNA‐binding SNPs for PC risk is absent.

Pancreatic cancer EWAS data‐mining should help us to perform a genome‐wide evaluation of the miRNA‐binding polymorphisms. The exome chip (Illumina Human Exome Beadchip) we previously used was one platform that primarily focused on variations in the exon regions of genes. Based on EWAS data, the genotypes of un‐assayed SNPs in 3′ untranslated regions (3′ UTRs) could be accurately imputed, in addition to those in the flanking exon and intron regions.

In this study, we integrated the data from the database miRNASNP v2.0 and our PC EWAS to genome‐widely screen miRNA‐binding polymorphisms for PC risk. Followed by an independent case‐control study and corresponding functional assays, we identified a PC‐associated variant rs3802266 affecting miR‐181a‐2‐3p binding to the gene *ZHX2*.

## 2 MATERIALS AND METHODS

### 2.1 Study participants

A two‐stage case‐control study was conducted to assess the associations between SNPs and PC risk. In Discovery Stage, we conducted the first‐phase association study according to the EWAS imputation data of 943 cases and 3908 controls. The enrolment and characteristics of EWAS population were described previously.^10^ Replication Stage consisted of 904 cases and 1805 controls that were recruited from different hospitals at Wuhan area. All subjects were unrelated Han Chinese. All patients were histopathologically confirmed PC without previous chemotherapy or radiotherapy, while cancer‐free controls came from community nutritional surveys in the same area during the same time period. With a written informed consent, about 1‐mL venous blood was obtained from each participant, part of which was included in our previous studies.^22^, ^23^, ^24^, ^25^ This study was carried out under the approval from the ethics committee of Tongji Medical College, Huazhong University of Science and Technology.

### 2.2 Selection of candidate SNPs

**Figure 1:**
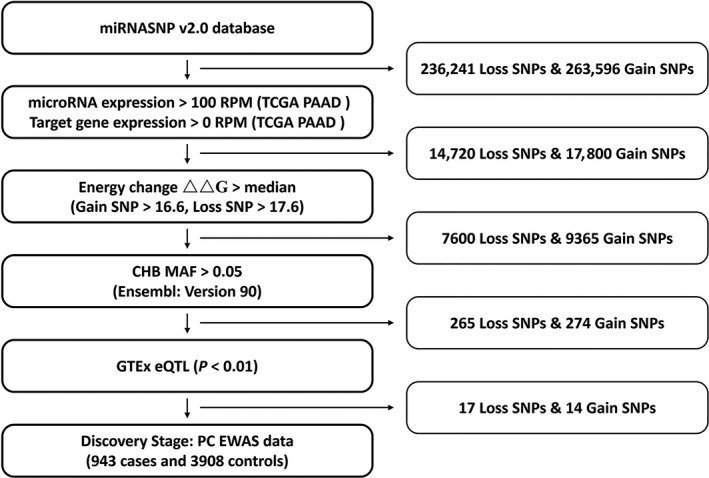
The flow chart of the integrative bioinformatics analysis in this study. Abbreviations: CHB, Han Chinese in Beijing, China; eQTL, expression quantitative trait loci; EWAS, exome‐wide association study; Gain SNPs, gain‐of‐function SNPs; GTEx, The Genotype‐Tissue Expression; Loss SNPs, loss‐of‐function SNPs; MAF, minor allele frequency; PAAD, pancreatic ductal adenocarcinoma; PC, pancreatic cancer; TCGA, The Cancer Genome Atlas

Through a systematic bioinformatics analysis, we screened out the most promisingly functional polymorphisms in miRNA‐binding sites for PC risk on a genome‐wide scale. Totally, there were 236 241 loss‐of‐function SNPs (Loss SNPs) and 263 696 gain‐of‐function SNPs (Gain SNPs) that predictably impact miRNA‐target interactions in the credible database miRNASNP v2.0 we previously built.^20^ First, we narrowed down candidate microRNA‐related polymorphisms according to the transcriptome data from The Cancer Genome Atlas (TCGA) pancreatic ductal adenocarcinoma (PAAD) data sets. We picked the SNPs whose affecting miRNAs that authentically expressed in PC tissues, with an expression level more than 100 reads per million mapped reads (RPM) according to the RNA‐seq data from TCGA database. We further exclude the SNPs with corresponding target genes that were not expressed in PC tissues. Second, given that the energy change (ΔΔG) represented the impact of a SNP on the binding between miRNA and mRNA, we selected SNPs with altered energy bigger than median ΔΔG (17.6 kcal/mol for Loss SNPs and 16.6 kcal/mol for Gain SNPs). Third, common variants with minor allele frequencies (MAFs) larger than 0.05 in Han Chinese in Beijing (CHB) were chosen for next‐step screening on the basis of data from Ensembl database (version 90: http://e90.ensembl.org/Homo_sapiens). Fourth, we sorted out the SNPs that were eQTLs (*P* < .01) whose gain‐of‐function allele was associated with lower expression of target gene or loss‐of‐function allele was associated with higher expression according to The Genotype‐Tissue Expression (GTEx) database,^26^ in the light of inhibition and decay effect of miRNA on mRNA. Finally, promising SNPs were submitted to a previous PC EWAS data set which was well genotype‐imputed as mentioned before.^10^ The process of the integrative bioinformatics analysis is summarized in Figure 1.

### 2.3 Genotyping

DNA were extracted from blood samples using RelaxGene Blood System DP319‐02 (Tiangen). In Replication Stage, the genotypes of promising SNP were determined through TaqMan SNP Genotyping Assays on the platform of 7900HT Fast Real‐Time PCR (Applied Biosystems). For quality control, we randomly selected 5% samples to assess the reproducibility and found out a concordance rate of 100%.

### 2.4 Cell lines and cell culture

PANC‐1 and BxPC‐3 cells were obtained from China Center for Type Culture Collection. Two cell lines were periodically authenticated by short tandem repeat (STR) and were checked for the absence of mycoplasma contamination (MycoAlert). Cells were cultured into Dulbecco's Modified Eagle's Medium (DMEM; Gibco), with addition of 10% foetal bovine serum (FBS; Gibco) and 1% antibiotics (100 U/mL penicillin and 100 μg/mL streptomycin), under the environment with an atmosphere of 5% CO^2^ and 37°C.

### 2.5 Construction of reporter plasmids, transient transfections and luciferase assays

The whole 1278 bp DNA fragment (chr8:123985478‐123986755) of the 3′ UTR of *ZHX2* (zinc fingers and homeoboxes 2), which contained the assumptive miRNA‐target binding site (TACTACTGCGTTTTCAA/GTGG), were synthesized and inserted into pmirGLO vector (Promega) in 5′ SacI and 3′ XhoI restrictive sites by Genewiz Company. The mimics of miR‐181a‐2‐3p (5′ to 3′, ACCACUGACCGUUGACUGUACC) and specific inhibitors were purchased from Gene Pharma Company. For assays, after PC cells were seeded into 96‐well plates, 50 ng constructed plasmids and 6 pmol miR‐181a‐2‐3p mimics and/or inhibitors were cotransfected using Lipofectamine 3000 Reagent (Invitrogen) following manufacturer's instruction. Forty‐eight hours later, Firefly and Renilla luciferase activities were detected by Dual Luciferase Reporter Assay System (Promega). The ratio of Firefly to Renilla luciferase activity was calculated as relative luciferase activity for each sample. Three independent assays were performed, while each assay was conducted in triplicate.

### 2.6 Statistical analysis

The distribution differences of gender, age and genotype between cases and controls were estimated by *t* test or chi‐square test whenever appropriate. The odds ratios (ORs) and corresponding 95% confidence intervals (95%CIs) were calculated through multivariate logistic regression, adjusted by gender and age. For statistical analyses, PLINK software was used in the discovery stage and SPSS Software v20.0 (SPSS, USA) was used in the replication stage. *P* values were two‐sided and the criterion of *P* < .05 was considered as statistically significant.

## 3 RESULTS

### 3.1 Population characteristics

Characteristics of the participants from Replication Stage were summarized in Table 1. In Replication Stage, 904 cases and 1805 controls were recruited, while cases and controls were well matched in terms of gender and age group (*P* > .05). We then combined the two data sets from both discovery and replication stage, and a total of 1847 cases and 5713 controls were included in the combined analyses (Table S1).

**Table 1:** The characteristics of the study population in Replication Stage

	Case No. (%)	Control No. (%)	*χ^2^*	*P*
Total	904	1805		
Gender				
Male	725 (80.2)	1410 (78.1)	1.57	2.11E‐01
Female	179 (19.8)	395 (21.9)		
Age, mean (SD)	60.1 (11.4)	60.4 (10.7)		5.75E‐01†
Age group				
<60	407 (45.0)	866 (48.0)	2.11	1.46E‐01
≧60	497 (55.0)	939 (52.0)		

Abbreviation: SD, standard deviation.

^†^ *P* value was calculated by the *t* test.

### 3.2 Selection of candidate SNPs and association analyses

As the results of the bioinformatics analysis on the data from miRNASNP v2.0, TCGA, Ensembl and GTEx, 31 common SNPs were selected as candidate SNPs for the first‐stage case‐control study which was our previous EWAS (Figure 1, Table S2).

Shown in Table 2, only rs3802266 was found to be significantly associated with PC susceptibility in Discovery Stage (additive model: OR = 1.23, 95%CI = 1.10‐1.38, *P* = 3.55E‐04). The SNP rs3802266 was then tested in Replication Stage, and positive results were successfully replicated in the additional sample set (AG vs AA: OR = 1.20, 95%CI = 1.01‐1.42, *P* = 3.76E‐03; dominant model: OR = 1.21, 95%CI = 1.03‐1.43, *P* = 1.90E‐02; additive model: OR = 1.17, 95%CI = 1.03‐1.33, *P* = 1.61E‐02; Table 3).

**Table 2:** Association between individual SNP and pancreatic cancer risk in Discovery Stage

SNP	Position	Cases	Controls	OR (95% CI)	*P*
		HW/HT/HV	HW/HT/HV		
rs529974	chr 1:20826910	844/97/2	3502/397/9	1.05 (0.83‐1.31)	7.03E‐01
rs9259	chr 1:25168124	285/478/165	1126/1978/727	0.96 (0.86‐1.06)	4.07E‐01
rs6547016	chr 2:75888160	328/470/145	1330/1926/652	0.96 (0.86‐1.07)	4.42E‐01
rs13396556	chr 2:240900097	594/314/35	2511/1211/186	1.01 (0.89‐1.15)	8.75E‐01
rs1127898	chr 3:33186356	289/473/181	1202/2053/653	1.07 (0.96‐1.19)	2.30E‐01
rs1044147	chr 4:763077	768/162/13	3173/700/35	1.01 (0.86‐1.20)	8.81E‐01
rs3733326	chr 4:57261234	545/333/60	2242/1415/220	1.01 (0.89‐1.13)	9.23E‐01
rs6844815	chr 4:90167781	277/465/201	1127/1990/789	1.01 (0.91‐1.12)	8.94E‐01
rs1298	chr 5:179289895	652/258/32	2747/1063/97	1.07 (0.93‐1.22)	3.54E‐01
rs2719236	chr 8:56924362	878/65/0	3667/231/2	1.17 (0.88‐1.56)	2.83E‐01
rs2290702	chr 8:71646980	830/112/1	3503/397/8	1.18 (0.94‐1.46)	1.48E‐01
**rs3802266**	**chr 8:123985708**	**424/429/79**	**1976/1617/252**	**1.23 (1.10‐1.38)**	**3.55E‐04**
rs730720	chr 10:73772762	824/118/1	3345/560/3	0.84 (0.68‐1.04)	1.16E‐01
rs1678623	chr 10:73821633	710/205/28	2863/973/72	0.94 (0.81‐1.09)	4.20E‐01
rs10832948	chr 11:18628730	367/450/126	1598/1766/544	1.03 (0.93‐1.15)	5.37E‐01
rs1060709	chr 13:31903834	353/456/134	1407/1889/612	0.95 (0.86‐1.06)	3.62E‐01
rs1051332	chr 13:52507720	371/436/135	1528/1850/527	1.02 (0.92‐1.14)	6.67E‐01
rs4785920	chr 16:3000016	344/446/153	1339/1923/646	0.95 (0.85‐1.05)	2.93E‐01
rs6944	chr 16:10622895	264/568/111	1071/2389/448	0.99 (0.87‐1.11)	8.01E‐01
rs2279875	chr 16:57610832	421/426/96	1805/1745/358	1.07 (0.96‐1.20)	2.35E‐01
rs3743599	chr 16:75646576	533/368/42	2355/1334/219	1.08 (0.96‐1.22)	1.95E‐01
rs1946482	chr 16:89762410	411/437/95	1681/1888/339	1.01 (0.91‐1.14)	8.04E‐01
rs11062	chr 17:1683012	267/460/216	1167/1897/844	1.06 (0.96‐1.17)	2.73E‐01
rs1582	chr 19:44830892	465/370/105	1979/1580/309	1.11 (0.99‐1.24)	6.82E‐02
rs1806940	chr 20:35945174	578/327/38	2389/1331/186	0.98 (0.86‐1.11)	7.19E‐01
rs1046612	chr 20:43996189	567/322/50	2322/1369/184	1.01 (0.90‐1.14)	8.50E‐01
rs747948	chr 20:60964301	732/198/13	3050/824/34	1.07 (0.91‐1.26)	4.00E‐01
rs5752330	chr 22:26859942	577/328/38	2350/1373/185	0.97 (0.85‐1.10)	6.11E‐01

Note

All ORs, 95%CIs and *P* values were adjusted by gender and age group. The nominal significant results were in bold.

Abbreviations: 95% CI, 95% confidence interval; HT, heterozygote; HV, variant homozygote; HW, wild‐type homozygote; OR, odds ratio; SNP, single nucleotide polymorphism.

**Table 3:** Association between rs3802266 and pancreatic cancer risk in Replication Stage and Combined Stage

	Replication stage				Combined stage			
	Cases (%)	Controls (%)	OR (95%CI)	*P*	Cases (%)	Controls (%)	OR (95%CI)	*P*
rs3802266								
AA	410 (46.5)	910 (51.4)	1.00		834 (46.0)	2886 (51.4)	1.00	
AG	399 (45.3)	740 (41.8)	**1.20 (1.01‐1.42)**	**3.76E‐02**	828 (45.7)	2357 (42.0)	**1.23 (1.10‐1.38)**	**2.56E‐04**
GG	72 (8.2)	120 (6.8)	1.33 (0.97‐1.82)	7.93E‐02	151 (8.3)	372 (6.6)	**1.41 (1.15‐1.74)**	**9.98E‐04**
Dominant model			**1.21 (1.03‐1.43)**	**1.90E‐02**			**1.26 (1.13‐1.40)**	**3.00E‐05**
Recessive model			1.22 (0.90‐1.65)	2.04E‐01			**1.28 (1.05‐1.56)**	**1.46E‐02**
Additive model			**1.17 (1.03‐1.33)**	**1.61E‐02**			**1.21 (1.11‐1.31)**	**1.29E‐05**

Note

All the *P* values were adjusted by gender and age group. The nominal significant results were in bold.

Abbreviations: 95% CI, 95% confidence interval; OR, odds ratio.

After two stages were pooled together, significant association between rs3802266 and PC risk was consistent in Combined Study (Table 3). Compared to those carrying the wild‐type genotype AA, individuals carrying AG genotype of rs3802266 had higher risk of PC (OR = 1.23, 95%CI = 1.10‐1.38, *P* = 2.56E‐04). A dominant model combining AG with GG into a G‐carrier group (AG plus GG) showed that the carriers of rs3802266‐A got an increased PC risk (OR = 1.26, 95%CI = 1.13‐1.40, *P* = 3.00E‐05). Similar result was seen in the additive model, with per‐G‐allele OR of 1.21 (95%CI = 1.11‐1.31, *P* = 1.29E‐04).

### 3.3 Dual luciferase reporter gene assays

**Figure 2:**
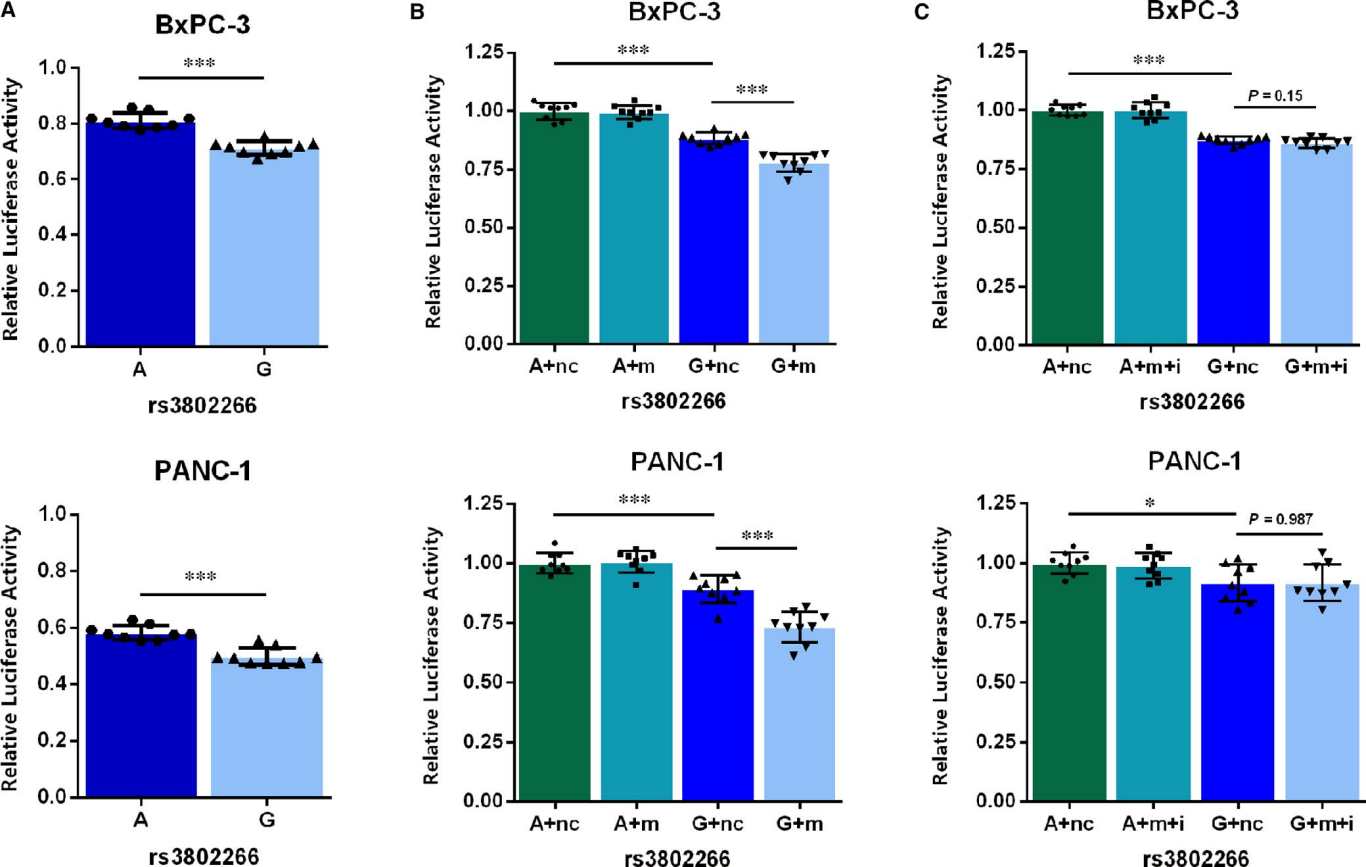
The effect of rs3802266 on miR‐181a‐2‐3p: *ZHX2* binding. A, Relative reporter gene activity from vectors pmirGLO bearing 3′UTR of *ZHX2* with the rs3802266‐A allele (pmirGLO‐rs3802266‐A) or rs3802266‐G allele (pmirGLO‐rs3802266‐G) in BxPC‐3 and PANC‐1 cells. B, Relative reporter gene activity of the pmirGLO‐rs3802266‐A and pmirGLO‐rs3802266‐G constructs cotransfected with miR‐181a‐2‐3p into BxPC‐3 and PANC‐1 cells. C, Relative reporter gene activity of the pmirGLO‐rs3802266‐A and pmirGLO‐rs3802266‐G constructs cotransfected with miR‐181a‐2‐3p and its specific inhibitor into BxPC‐3 and PANC‐1 cells. Firefly luciferase/Renilla luciferase were calculated and normalized to blank or corresponding negative controls as relatively luciferase activity. Results were shown as means ± SD from three experiments, each with three replicates. Differences between groups were analysed using two‐sided *t* tests. *P < .05, **P < .01, ***P < .001. Abbreviations: A, pmirGLO‐rs3802266‐A; G, pmirGLO‐rs3802266‐G; i, inhibitors of miR‐181a‐2‐3p; m, miR‐181a‐2‐3p; nc, negative control

We conducted dual luciferase reporter assays to investigate the effect of rs3802266 on the binding between miR‐181a‐2‐3p and *ZHX2* in PANC‐1 and BxPC‐3 cell lines. Compared to rs3802266‐A, the constructed plasmids containing rs3802266‐G demonstrated 14.4% and 12.0% reduction of luciferase activity in the absence of miR‐181a‐2‐3p mimics in PANC‐1 and BxPC‐3 cells, respectively (Figure 2, *P* < .001). After transient cotransfection with miR‐181a‐2‐3p mimics, a more significant reduction in luciferase levels was observed in the G‐allele subgroup, approximately 26.9% in PANC‐1 and 22.2% in BxPC‐3 cells (*P* < .001). While specific inhibitors were additionally transfected into PC cells, miR‐181a‐2‐3p inhibitors obviously reversed the repression of luciferase activity for rs3802266‐G plasmids. These findings revealed that rs3802266 A>G could influence the binding between miR‐181a‐2‐3p and the *ZHX2* 3′UTR.

### 3.4 Expression quantitative trait loci (eQTL) analyses

**Figure 3:**
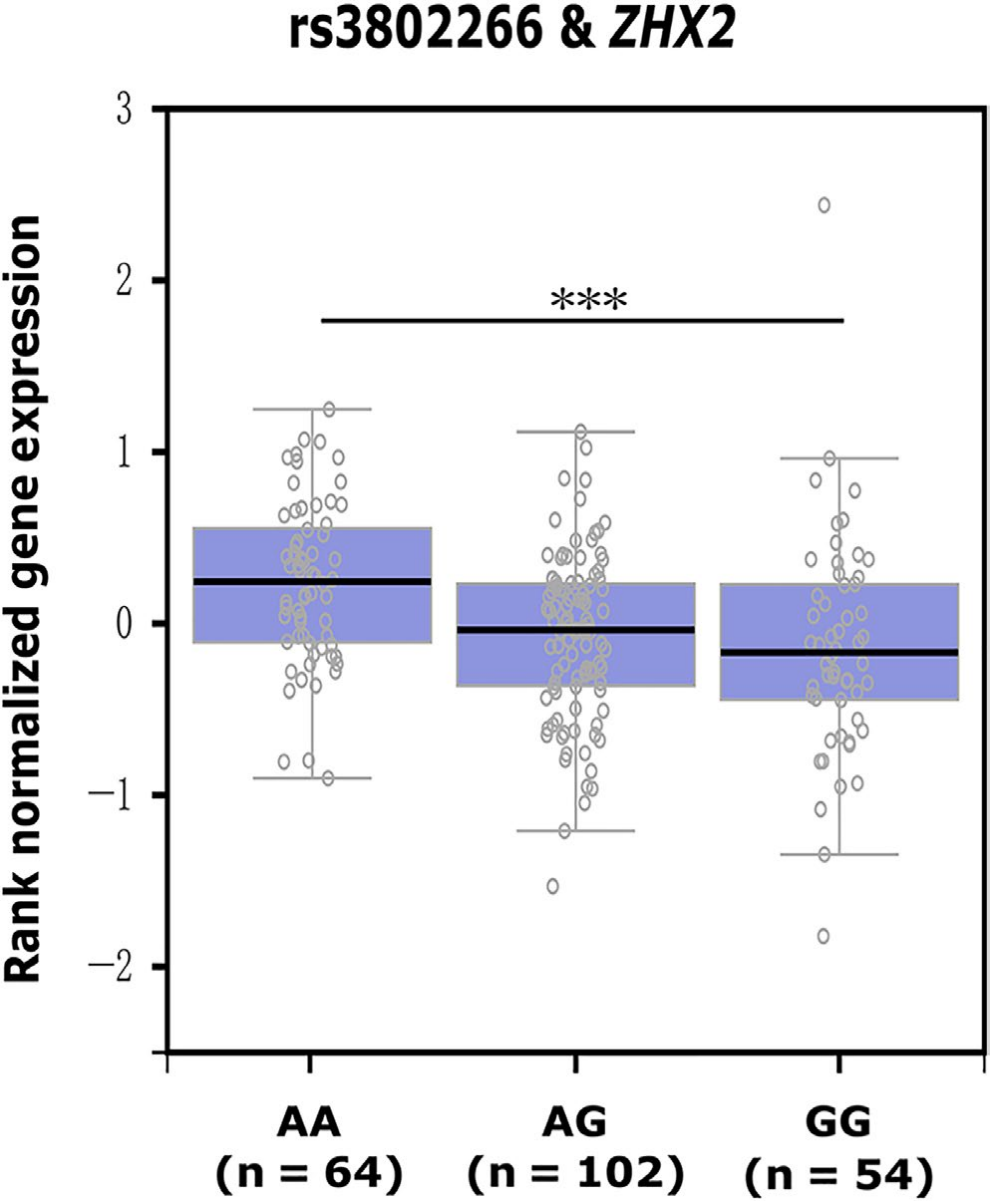
eQTL analyses on rs3802266 and *ZHX2* in pancreas tissues based on GTEx data. ***P < .001

To further investigate whether the functional variant rs3802266 affect *ZHX2* expression, we performed cis‐eQTL analysis using newly released data from GTEx (GTEx Analysis Release V7).^26^ It showed that individuals carrying the rs3802266‐G genotype had significantly lower *ZHX2* mRNA levels in pancreas tissue samples compared with those carrying the rs3802266‐A genotype (Figure 3). On the other side, a huge‐scale eQTL analysis containing 31‐684 blood samples, named eQTLGen, also indicated rs3802266 as a significant cis‐eQTL of *ZHX2* (*P* = 1.72E‐118).^27^

## 4 DISCUSSION

In this study, we systematically evaluated the associations between common genetic variants in microRNA‐binding sites and Chinese PC risk on a genome‐wide scale. With the excavation of the miRNASNP database and PC EWAS data, we conducted miRNA‐related bioinformatics analysis, performed association studies with a two‐stage design and characterized the SNP's functionality via molecular biology experiments. Taken together, we highlighted a PC‐associated SNP rs3802266 at 8q24.13 in total 1847 cases and 5713 controls. Located in 3′UTR of the gene *ZHX2*, the minor rs3802266‐G allele created a stronger binding site for miR‐181a‐2‐3p, lowered the expression of *ZHX2* in vivo and raised PC risk in Chinese population.

Considerable evidences supported that the gene *ZHX2* might be important to PC. Belonging to zinc fingers and homeoboxes gene family that were nuclear homodimeric transcriptional repressors,^28^ *ZHX2* was implicated in various human diseases, such as podocyte disease,^29^ multiple myeloma^30^ and hepatocellular carcinoma (HCC).^31^, ^32^, ^33^ In particular, *ZHX2* was found to inhibit HCC proliferation in vitro and in vivo by reducing the expression of Cyclins A and E, with a lower nuclear level in HCC samples.^34^ It highly suggested the tumour‐suppressing role that *ZHX2* played in carcinogenesis.

On the other hand, miR‐181 family were indicated to be evolutionarily conserved across vertebrates, which implied their functional importance.^35^ Several studies indicated the involvement of miR‐181a in different cellular processes (growth, proliferation, death and survival) and even carcinogenesis.^36^, ^37^ Overexpression of miR‐181a might facilitate cancer metastasis and invasion.^38^, ^39^ Moreover, miR‐181a was found consistently and highly expressed in PC cell lines, and down‐regulated miR‐181a expression could inhibit proliferation and migration of PC cells.^40^ Above all, it supported that miR‐181a‐2‐3p was an oncogenic miRNA in PC.

Therefore, we speculated that the functional SNP rs3802266 facilitated the binding between miR‐181a‐2‐3p and *ZHX2*, reduced expression of the potential anti‐oncogene *ZHX2*, and thus promoted PC occurrence and development. While our study reported a systematic screening and evaluation of the miRNA‐binding‐site polymorphisms for PC, some limitations should be acknowledged. First, more functional analyses were warranted to uncover the role of *ZHX2* and miR‐181a‐2‐3p in PC aetiology. Second, we only focused on variants in 3′UTRs of genes and ignored genetic variants residing in other regulatory elements such as promoters, enhancers and silencers. Third, we selected SNPs based on TCGA data primarily came from Caucasians, so we might overlook some Chinese‐specific variants in our study focusing on Chinese population. Forth, insufficient demographic data such as family history, diet, smoking and drinking, prevented us to execute a more accurate adjustment in statistical analyses.

In summary, we highlighted a functional polymorphism rs3802266 affecting miR‐181a‐2‐3p binding to *ZHX2* via a genome‐wide evaluation of the variants in miRNA‐binding sites for PC risk. The research strategy integrating reliable bioinformatics tools, existing GWAS/EWAS data and molecular functional assays, could be helpful to expand and deepen the understanding of genetic aetiology for phenotypes and diseases.

## CONFLICT OF INTEREST

All authors declare no conflicts of interest.

## AUTHOR CONTRIBUTIONS

JK drafted the manuscript. JK and XP analysed and interpreted the data. JK performed the functional experiments. SM, JT, PY, XW, DZ, YY, YZ and YG collected the samples. JG, RZ, JC and XM contributed reagents/materials/analysis tools. JK and XM designed the study. All authors have read the manuscript and approved the submission.

## Data Availability

The data sets during the current study are available from the corresponding author on reasonable request.
