# Supplementary material for: Evaluation of polymorphisms in microRNA‐binding sites and pancreatic cancer risk in Chinese population
Source: J Cell Mol Med. 2019 Dec 27;24(3):2252–9. doi: 10.1111/jcmm.14906 (PMC7011162; doi:10.1111/jcmm.14906)
Supplement: Supplementary file 1 [file JCMM-24-2252-s001.docx]

Table S1. The characteristics of the study population in Discovery Stage and Combined Stage.

|  | Discovery Stage | |  | Combined Stage | |
| --- | --- | --- | --- | --- | --- |
|  | Case (%) | Control (%) |  | Case (%) | Control (%) |
| Total | 943 | 3908 |  | 1847 | 5713 |
| Gender |  |  |  |  |  |
| Male | 748 (79.3) | 2455 (62.8) |  | 1473 (79.8) | 3865 (67.7) |
| Female | 195 (20.7) | 1453 (37.2) |  | 374 (20.2) | 1848 (32.3) |
| Age, mean (SD) | 60.2 (11.6) | 63.7 (10.2) |  | 60.2 (11.6) | 62.6 (10.5) |
| Agegroup |  |  |  |  |  |
| < 60 | 449 (45.8) | 712 (35.8) |  | 835 (45.2) | 2189 (38.3) |
| ≧60 | 532 (54.2) | 1279 (64.2) |  | 1012 (54.8) | 3524 (61.7) |

Abbreviations: SD, standard deviation.

Table S2. Summary of candidate SNPs obtained from the bioinformatics analysis.

| SNP | Position | CHB MAF | Gene | TCGA Exp | eQTL *P* | microRNA | TCGA Exp | Effect | Energy change |
| --- | --- | --- | --- | --- | --- | --- | --- | --- | --- |
| rs529974 | Chr 1: 20826910 | 11.0% | *MUL1* | 931.37 | 1.60E-04 | hsa-miR-342-3p | 110.28 | Gain | 20.4 |
| rs9259 | Chr 1: 25168124 | 52.6% | *CLIC4* | 4900.85 | 3.70E-04 | hsa-miR-574-3p | 104.62 | Gain | 21.2 |
| rs6547016 | Chr 2: 75888160 | 34.1% | *MRPL19* | 880.41 | 2.30E-04 | hsa-miR-542-3p | 230.39 | Gain | 18.9 |
| rs13396556 | Chr 2: 240900097 | 25.6% | *NDUFA10* | 1617.28 | 6.60E-06 | hsa-miR-103a-3p | 12413.42 | Gain | 24.2 |
| rs8369 | Chr 2: 240900227 | 13.4% | *NDUFA10* | 1617.28 | 1.60E-15 | hsa-miR-15a-5p | 102.07 | Gain | 21.7 |
|  |  |  |  |  |  | hsa-miR-15b-5p | 122.12 | Gain | 20.6 |
|  |  |  |  |  |  | hsa-miR-16-5p | 236.5 | Gain | 24.9 |
|  |  |  |  |  |  | hsa-miR-424-5p | 113.8 | Gain | 18.2 |
| rs1127898 | Chr 3: 33186356 | 57.3% | *CRTAP* | 3866.52 | 2.90E-04 | hsa-miR-21-3p | 2421.83 | Loss | 23.2 |
| rs1044147 | Chr 4: 763077 | 12.2% | *PCGF3* | 1406.7 | 1.00E-05 | hsa-miR-141-3p | 796.89 | Loss | 19.3 |
|  |  |  |  |  |  | hsa-miR-200a-3p | 542.06 | Loss | 19.5 |
| rs3733326 | Chr 4: 57261234 | 19.5% | *PPAT* | 269.69 | 8.20E-03 | hsa-miR-182-5p | 8516.93 | Gain | 20.3 |
| rs6844815 | Chr 4: 90167781 | 46.3% | *GPRIN3* | 235.77 | 1.30E-08 | hsa-miR-338-3p | 971.25 | Loss | 19.6 |
| rs1298 | Chr 5: 179289895 | 22.0% | *TBC1D9B* | 3218.29 | 1.10E-03 | hsa-miR-486-5p | 132.08 | Gain | 27.2 |
| rs1045251 | Chr 6: 30259657 | 37.8% | *HCG18* | 400.53 | 2.30E-10 | hsa-miR-24-3p | 1362.83 | Loss | 20.9 |
| rs2719236 | Chr 8: 56924362 | 8.5% | *LYN* | 1421.37 | 3.20E-03 | hsa-miR-194-5p | 4758.46 | Loss | 18 |
| rs2290702 | Chr 8: 71646980 | 7.3% | *XKR9* | 83 | 4.60E-30 | hsa-miR-199a-3p | 4696.07 | Loss | 19.8 |
|  |  |  |  |  |  | hsa-miR-199b-3p | 4685.25 | Loss | 19.8 |
| rs3802266 | Chr 8: 123985708 | 26.8% | *ZHX2* | 1144.25 | 1.10E-04 | hsa-miR-181a-2-3p | 830.44 | Gain | 21.1 |
| rs730720 | Chr 10: 73772762 | 9.8% | *CHST3* | 907.92 | 2.30E-07 | hsa-miR-183-5p | 2875.46 | Gain | 22.2 |
| rs1678623 | Chr 10: 73821633 | 11.0% | *SPOCK2* | 1500.59 | 1.00E-05 | hsa-miR-361-3p | 144.71 | Loss | 30.3 |
| rs10832948 | Chr 11: 18628730 | 30.5% | *SPTY2D1* | 946.31 | 3.40E-03 | hsa-miR-132-3p | 186.06 | Gain | 23.9 |
| rs1060709 | Chr 13: 31903834 | 42.7% | *B3GALTL* | 256.78 | 1.00E-05 | hsa-miR-125a-5p | 682.62 | Gain | 22.2 |
|  |  |  |  |  |  | hsa-miR-125b-5p | 1148.27 | Gain | 22.3 |
| rs1051332 | Chr 13: 52507720 | 36.6% | *ATP7B* | 381 | 3.70E-05 | hsa-miR-30d-5p | 4895.46 | Loss | 18.2 |
|  |  |  |  |  |  | hsa-miR-30e-5p | 4229.84 | Loss | 18.3 |
| rs4785920 | Chr 16: 3000016 | 46.3% | *FLYWCH1* | 699.22 | 3.40E-06 | hsa-miR-361-3p | 144.71 | Loss | 31.6 |
| rs6944 | Chr 16: 10622895 | 57.3% | *EMP2* | 2480.24 | 2.50E-13 | hsa-miR-181a-5p | 2264.89 | Loss | 23.4 |
|  |  |  |  |  |  | hsa-miR-181b-5p | 517.74 | Loss | 20.1 |
| rs16955473 | Chr 16: 55618667 | 12.2% | *LPCAT2* | 1012.06 | 2.30E-05 | hsa-miR-21-3p | 2421.83 | Gain | 18 |
| rs2279875 | Chr 16: 57610832 | 30.5% | *GPR114* | 108.33 | 3.90E-04 | hsa-miR-146a-5p | 115.98 | Loss | 24.2 |
|  |  |  |  |  |  | hsa-miR-146b-5p | 698.05 | Loss | 23.3 |
| rs3743599 | Chr 16: 75646576 | 78.8% | *ADAT1* | 231.75 | 1.90E-04 | hsa-miR-17-3p | 227.64 | Loss | 24.3 |
| rs1946482 | Chr 16: 89762410 | 25.6% | *CDK10* | 1375.68 | 1.60E-04 | hsa-miR-150-5p | 1118.22 | Loss | 20.2 |
| rs11062 | Chr 17: 1683012 | 37.8% | *SMYD4* | 233.41 | 2.40E-06 | hsa-miR-27a-3p | 1263.52 | Gain | 20.7 |
|  |  |  |  |  |  | hsa-miR-27b-3p | 1502.78 | Gain | 17.8 |
| rs1582 | Chr 19: 44830892 | 75.6% | *ZFP112* | 117.13 | 3.70E-11 | hsa-miR-106b-5p | 122.43 | Gain | 25.9 |
|  |  |  |  |  |  | hsa-miR-20a-5p | 182.4 | Gain | 22.6 |
|  |  |  |  |  |  | hsa-miR-93-5p | 2847.69 | Gain | 21.4 |
| rs1806940 | Chr 20: 35945174 | 81.7% | *MANBAL* | 1237.98 | 7.60E-06 | hsa-miR-150-5p | 1118.22 | Loss | 21.1 |
| rs1046612 | Chr 20: 43996189 | 30.0% | *SYS1* | 1008.58 | 1.10E-11 | hsa-miR-141-3p | 796.89 | Loss | 23.4 |
|  |  |  |  |  |  | hsa-miR-200a-3p | 542.06 | Loss | 27.4 |
| rs747948 | Chr 20: 60964301 | 13.4% | *CABLES2* | 210.58 | 1.80E-10 | hsa-miR-127-5p | 428.98 | Loss | 24.2 |
| rs5752330 | Chr 22: 26859942 | 73.2% | *HPS4* | 889.09 | 1.30E-05 | hsa-miR-29a-3p | 5563.51 | Loss | 22.2 |
|  |  |  |  |  |  | hsa-miR-29b-3p | 579.99 | Loss | 27 |
|  |  |  |  |  |  | hsa-miR-29c-3p | 1734.71 | Loss | 23.9 |

Abbreviations: SNP, single nucleotide polymorphism; CHB, Han Chinese in Bejing, China; MAF, minor allele frequency; Exp, expression; eQTL, expression quantitative trait loci.
